# Positive Affect and Academic Skill Development Through ChatGPT in Higher Education

**DOI:** 10.3390/ejihpe16070100

**Published:** 2026-07-13

**Authors:** Bonginkosi A. Thango, Lerato Matshaka, Alaa M. S. Azazz, Ibrahim A. Elshaer

**Affiliations:** 1 Department of Electrical and Electronic Engineering Technology, University of Johannesburg, Johannesburg 2092, South Africa; 2 Department of Nursing Science, University of Johannesburg, Johannesburg 2092, South Africa; loratom@uj.ac.za; 3 Social Studies Department, College of Art, King Faisal University, Al-Ahsa 31982, Saudi Arabia; aazazz@kfu.edu.sa; 4 Department of Management, School of Business, King Faisal University, Al-Ahsa 31982, Saudi Arabia

**Keywords:** positive affect, academic skill development, ChatGPT, attitude, learning orientation, mediation, moderation, Broaden-and-Build, PLS-SEM, higher education

## Abstract

This study investigates how positive affect during ChatGPT-4 use is associated with perceived academic skill development in higher education, emphasising the mediating role of attitudes toward ChatGPT and the moderating role of learning orientation, grounded in Broaden-and-Build Theory and the Technology Acceptance Model. The enrichment of academic skills through ChatGPT is proposed to be maximised when students who experience positive affect develop positive attitudes toward the tool and channel their interactions through deep learning orientations. This study uses quantitative analysis with n = 12,035 active ChatGPT-using students from 135 countries. PLS-SEM using bootstrapping with 200 resamples is employed for data analysis. The results confirm that positive affect is positively associated with perceived academic skill development (H1: β = 0.199, *p* < 0.001) and attitude (H2: β = 0.267, *p* < 0.001), and that attitude is significantly associated with skill development (H3: β = 0.354, *p* < 0.001). Attitude partially mediates the positive affect to skill development relationship (H4: β = 0.095, t = 24.923, *p* < 0.001). Learning orientation negatively moderates both the attitude skill development (H5: β = −0.021) and positive affect skill development (H6: β = −0.045) pathways, indicating a desirable difficulty effect: when learning orientation is high, less evaluative scaffolding is needed for skill acquisition, consistent with cognitive challenge theory. What this implies for practice is that access alone is not enough. Institutions need to cultivate AI interactions that feel emotionally positive and, alongside them, a learning-oriented form of engagement; only when the two are developed together does ChatGPT’s potential for academic skill development carry across the diverse global contexts of higher education.

## 1. Introduction

Conversational generative AI has moved into higher education quickly, and in doing so it has changed the everyday work of students. Learners now turn to these tools to look up explanations, sketch out ideas, revise drafts, sharpen their reasoning, and pick up the competencies particular to their disciplines. ChatGPT sits at the centre of this shift, largely because the help it offers is immediate, conversational, and tailored to whatever task is in front of the student. A good deal has already been written about how students perceive ChatGPT, how readily they accept AI tools, the ethical worries they raise, and what generative AI means for teaching, assessment, and academic integrity (Nahiyan & Rahman, 2026; Verma et al., 2026; Ronaghi & Ronaghi, 2026; Gençel et al., 2026). This work establishes fairly convincingly that ChatGPT can support academic tasks. What it has not pinned down is the psychological route by which a student’s interaction with the tool actually turns into skill. Most studies frame ChatGPT use as a cognitive, behavioural, or technological matter, and they tend to picture the student as someone weighing up usefulness, convenience, risk, and intention to use. Far less often is the student treated as an emotionally situated learner whose feelings during the interaction colour how they engage with what the tool produces. This is a real gap, because students plainly do not approach ChatGPT through cold calculation alone. They feel curious, excited, enthusiastic, and at times more confident while they work with it (Yao & Liu, 2026), and those feelings may be exactly what decides whether a student probes a prompt further, reads the output with a critical eye, sticks with a hard task, and in the end converts the exchange into stronger research, writing, and analytical ability.

The present study addresses this gap by examining how positive affect during ChatGPT use is associated with perceived academic skill development among higher education students. Positive affect is conceptualised as favourable emotional engagement with ChatGPT, including curiosity, excitement, enthusiasm, engagement, and positive emotional valence. Academic skill development refers to students’ perceived improvements in research skills, critical thinking, writing, analytical ability, and field-specific competencies. The study further examines attitude toward ChatGPT as a mediating mechanism and learning orientation as a moderating condition. In this model, attitude captures students’ evaluative orientation toward ChatGPT as useful, effective, and beneficial for academic purposes, while learning orientation reflects students’ intrinsic motivation to master skills, deepen understanding, and use academic challenges as a pathway to growth. The theoretical foundation of the study is drawn from Broaden-and-Build Theory, the Technology Acceptance Model, and desirable difficulty theory. Broaden-and-Build Theory suggests that positive emotions expand learners’ thought–action repertoires and help build durable intellectual and psychological resources (Fredrickson, 2001). Brought into the context of ChatGPT-mediated learning, the implication is that the student who feels good during the interaction is the one more inclined to chase down alternative explanations, work back and forth with prompts, weigh the output critically, and through that effort build academic skill. The Technology Acceptance Model supplies the evaluative half of the picture, accounting for the way a positive experience crystallises into a favourable attitude toward the technology, which in turn feeds more productive engagement and better outcomes (Davis, 1989). Desirable difficulty theory adds a boundary condition by suggesting that skill development may be strongest when students engage with productive cognitive challenges rather than relying only on smooth or affectively positive experiences (Bjork & Bjork, 2011).

This study therefore proposes that positive affect is associated with perceived academic skill development both directly and indirectly through attitudes toward ChatGPT. Students who feel positive during ChatGPT use may develop stronger academic skills because positive affect promotes exploratory engagement, persistence, and confidence. At the same time, positive affect may first shape students’ attitudes toward ChatGPT, and these favourable evaluations may then support sustained skill-building engagement. However, the strength of these pathways may depend on students’ learning orientation. Students with strong learning orientation may require less affective or evaluative scaffolding because their intrinsic motivation to master academic tasks enables them to benefit from ChatGPT even when positive emotions or favourable attitudes are less pronounced. This possibility introduces a theoretically important moderation mechanism into the AI-mediated learning literature. The empirical analysis is based on a large international sample of 12,035 active ChatGPT-using higher education students from 135 countries. Partial least squares structural equation modelling is used to estimate the direct, mediated, and moderated pathways linking positive affect, attitude, learning orientation, and academic skill development. The results show that positive affect significantly predicts perceived academic skill development (β = 0.199, *p* < 0.001) and attitude toward ChatGPT (β = 0.267, *p* < 0.001). Attitude is the strongest direct predictor of perceived academic skill development (β = 0.354, *p* < 0.001), confirming the importance of students’ evaluative orientation toward ChatGPT. The mediation analysis further shows that attitude partially mediates the relationship between positive affect and perceived academic skill development (β = 0.095, *p* < 0.001). The model explains a substantial proportion of variance in perceived academic skill development, achieving R^2^ = 0.542.

A novel finding is that learning orientation negatively moderates both the attitude to perceived academic skill development pathway (β = −0.021, *p* < 0.001) and the positive affect to perceived academic skill development pathway (β = −0.045, *p* < 0.001). This indicates a desirable difficulty pattern where students with stronger learning orientation depend less on affective and evaluative support to achieve perceived academic skill development. Rather than suggesting that positive affect and attitudes are unimportant, this result shows that their effects are strongest among students who require greater affective and evaluative scaffolding. For highly learning-oriented students, perceived academic skill development appears to be more strongly associated with intrinsic mastery motivation and tolerance for cognitive challenge. This study is guided by three research questions:

- RQ1: How is positive affect during ChatGPT use associated with perceived academic skill development among higher education students?
- RQ2: Does attitude toward ChatGPT mediate the relationship between positive affect and academic skill development?
- RQ3: Does learning orientation moderate the affective and evaluative pathways through which ChatGPT use supports academic skill development?

The findings demonstrate that positive emotional engagement with ChatGPT is not merely an incidental feature of AI use, but an important psychological condition for perceived academic skill development. They also show that attitude toward ChatGPT operates as a key evaluative mechanism through which positive affect is associated with skill development. Most importantly, the results show that learning orientation functions as a boundary condition, revealing that students who are strongly motivated to master academic tasks may benefit from ChatGPT through more self-directed and cognitively challenging pathways.

### 1.1. Novelty of the Proposed Work

Although generative AI tools such as ChatGPT are now widely used in higher education, existing studies have largely focused on adoption, perceived usefulness, ethical concerns, academic integrity, writing support, and general learning engagement (Hajam & Jan, 2026; Moeiniasl, 2026; Pretorius & Thewissen, 2026; Connell Pensky et al., 2026). Comparatively less attention has been given to the affective and motivational mechanisms through which ChatGPT use contributes to academic skill development. Prior research has tended to examine whether students use ChatGPT, how they perceive it, or whether they consider it helpful, but has not sufficiently explained how positive emotional experiences during AI interactions become translated into research, writing, analytical, and field-specific skill gains.

This study advances the literature by developing an affective–evaluative model of ChatGPT-mediated academic skill development. Within this model, positive affect sits upstream as the driver of academic skill development, while attitude toward ChatGPT does the connecting work as the central mediating mechanism. This moves beyond conventional technology acceptance explanations by showing that students’ emotional experiences of ChatGPT use can shape their evaluative orientation and, through this pathway, their academic skill outcomes. By integrating Broaden-and-Build Theory with the Technology Acceptance Model, this study provides a psychologically richer account of how the benefits of AI-supported learning emerge.

A further novelty lies in the introduction of learning orientation as a moderating condition. The study does not assume that positive affect and favourable attitude operate uniformly across all students. Instead, it tests whether students with stronger learning orientation depend less on affective and evaluative scaffolding when developing academic skills through ChatGPT. The negative moderation effects provide evidence of a desirable difficulty pattern: for highly learning-oriented students, skill development appears to be supported by cognitive challenge, mastery motivation, and self-directed effort rather than by affective or evaluative amplification alone. This shows that the pathways to perceived academic skill development are not identical for all learners, offering a new explanatory perspective for AI-enhanced learning theory and practical guidance for designing differentiated AI pedagogies.

Finally, this study is distinctive in its outcome focus, concentrating on perceived academic skill development, that is, students’ perceived improvements in research, writing, analytical, critical thinking, and field-specific competencies, rather than on broad or undifferentiated tool use. This outcome is of growing concern for institutions interested in whether AI tools support durable academic capabilities. The empirical and methodological features that ground these relationships, including the large international sample and the analytical approach, are set out among the contributions below.

### 1.2. Research Contributions

Building on the novelty outlined above, the main contributions of this study can be summarised as follows:

- Theoretical contribution: an affective–evaluative framework that integrates Broaden-and-Build Theory, the Technology Acceptance Model, and desirable difficulty theory to explain how emotional, evaluative, and motivational mechanisms jointly shape ChatGPT-mediated academic skill development;
- Empirical contribution: evidence that positive affect predicts perceived academic skill development both directly and indirectly, with attitude toward ChatGPT identified as the key mediating mechanism and the strongest direct predictor of the outcome;
- Boundary condition contribution: identification of learning orientation as a negative moderator of both the affective and evaluative pathways, interpreted as a desirable difficulty effect in which highly learning-oriented students require less affective and evaluative scaffolding;
- Methodological contribution: application of PLS-SEM to a large international sample of 12,035 active ChatGPT-using higher education students from 135 countries, with the model explaining a substantial proportion of variance in academic skill development (R^2^ = 0.542);
- Practical contribution: guidance for higher education institutions indicating that effective AI implementation should cultivate positive affective experiences, favourable attitudes, and learning-oriented engagement rather than focusing only on technical access to ChatGPT.

The remainder of this paper is organised as follows. Section 2 presents the theoretical background and develops the hypotheses. Section 3 describes the research design, data source, sample, construct operationalisation, and analytical approach. Section 4 discusses the measurement model, reliability, validity, model fit, and structural model results. Section 5 presents the hypothesis testing, mediation, and moderation findings. Section 6 discusses the theoretical and practical meaning of the results. Section 7 concludes with implications, limitations, and directions for future research.

## 2. Theoretical Background and Hypothesis Development

Generative artificial intelligence has become a major focus in higher education research because tools such as ChatGPT are increasingly used for explanation, writing support, idea generation, revision, feedback, problem solving, and discipline-specific learning. Recent studies show that students use ChatGPT across diverse educational contexts, including medical education, English medium instruction, academic writing, physical therapy education, language learning, STEM education, and higher-order thinking development (Xu et al., 2026; Webb et al., 2026; El-Sobkey et al., 2026; Ayanwale & Omeh, 2026; Zheng et al., 2026). This literature confirms that ChatGPT can support academic work, but it also shows that the benefits of AI use depend on how students engage with the tool, how they evaluate it, and whether they use it as a learning resource rather than as a substitute for academic effort. Within this broader body of literature, academic skill development has become an increasingly important outcome. ChatGPT is not only used to complete isolated academic tasks. It may also support the development of research skills, writing quality, critical thinking, analytical reasoning, metacognitive awareness, and field-specific competence when students interact with it actively and critically (Begmatova & Saydazimova, 2026; Bauer et al., 2026; Alyasin & Shah, 2026; Zhang & Chen, 2026). However, the psychological mechanisms that explain why some students derive stronger skill development benefits from ChatGPT remain insufficiently understood. In particular, limited attention has been given to the affective and evaluative pathways through which positive emotions during ChatGPT use are translated into academic skill gains. This study addresses this gap by integrating Broaden-and-Build Theory, the Technology Acceptance Model, and desirable difficulty theory into a single framework. Positive affect is positioned as the upstream emotional driver of academic skill development. Attitude toward ChatGPT is positioned as the mediating evaluative mechanism through which affective experience becomes a more stable orientation toward productive AI use. Learning orientation is positioned as a moderating condition that may weaken the dependence of academic skill development on affective and evaluative scaffolding. The model therefore explains not only whether positive affect matters, but also how and under what learning conditions it contributes to academic skill development.

### 2.1. Broaden-and-Build Theory Applied to ChatGPT-Enhanced Skill Development

Broaden-and-Build Theory is the main affective anchor of this study. Its central claim is that positive emotions widen the momentary range of thoughts and actions available to a person and, over time, help them to accumulate lasting intellectual, psychological, and social resources (Fredrickson, 2001). Read against ChatGPT-mediated learning, the idea is fairly simple: a student who feels curious, excited, enthusiastic, confident, or simply engaged while using ChatGPT tends to be readier to try several explanations, test prompts, line up outputs against one another, rework responses, and keep an iterative exchange going. Behaviours like these matter for skill development precisely because they demand active processing rather than the passive reading of AI-generated text. Recent work in AI education lends weight to this affective reasoning. Studies of generative AI in academic writing report gains in writing quality, the felt experience of writing, semantic complexity, cohesion, lexical diversity, and confidence, provided that students use the tools within guided or reflective routines (Begmatova & Saydazimova, 2026; Bauer et al., 2026; Papanastasiou et al., 2026). Research focusing specifically on affective engagement adds that generative AI can heighten inspiration, attention, and determination, though these states rise and fall across the stages of writing and hinge on prompt design, the quality of feedback, and what the learner expected going in (Q. Q. Chen et al., 2026a; Zhao & Wang, 2026). The takeaway is that affect is not a side detail of ChatGPT use; it shapes how a student engages, how carefully they read what the tool returns, and whether the exchange turns out to be academically useful. For this reason, positive affect is treated here as an enabling condition for academic skill development. Students who find ChatGPT stimulating and emotionally supportive are the ones likely to stay with hard tasks, ask follow-up questions, treat outputs as raw material for refinement, and build competencies they can carry elsewhere. This reasoning underpins the direct pathway proposed from positive affect to academic skill development.

**H1.**  *Positive affect during ChatGPT use is positively associated with perceived academic skill development.*

### 2.2. Technology Acceptance Model and Attitudes Toward ChatGPT

On the evaluative side, the study leans on the Technology Acceptance Model. The model explains technology use through students’ perceptions of usefulness, ease of use, and their resulting attitudes toward the technology (Davis, 1989). In higher education, attitudes toward ChatGPT reflect students’ overall evaluative orientation toward the tool as useful, effective, beneficial, and appropriate for academic purposes. Students who develop favourable attitudes are more likely to engage with ChatGPT repeatedly, interpret its outputs constructively, and integrate it into academic work in ways that support learning rather than the replacement of effort. Recent studies confirm the relevance of acceptance-based explanations in generative AI education. Rizun et al. (2026) integrated the Technology Acceptance Model with ethical and behavioural frameworks and found that students’ adoption of ChatGPT is shaped by perceived control, social influence, trust-related factors, and performance evaluations. Alotaibi (2026) showed that attitude, subjective norms, and perceived behavioural control are major predictors of AI tool adoption among university students, while intention to use AI is linked to critical thinking engagement. Similarly, El-Sobkey et al. (2026) found that ChatGPT users reported stronger perceived usefulness and ease of use than non-users in physical therapy education. Ali et al. (2026) further showed that learning value, perceived credibility, and perceived intelligence shape attitudes toward ChatGPT, while attitudes predict intention to use it. These studies indicate that attitude is not merely a general opinion about ChatGPT. It is a cognitive evaluative mechanism that links students’ experiences of the tool to subsequent engagement and outcomes. In the present framework, attitude is expected to serve two functions. First, it is expected to be shaped by positive affect because emotionally positive interactions provide evaluative evidence that ChatGPT is useful and supportive. Second, it should help predict academic skill development, since a favourable attitude is what keeps a student engaging with the tool in a sustained, reflective, and purposeful way.

### 2.3. Positive Affect and Academic Skill Development

Within this study, positive affect during ChatGPT use means the favourable emotional states a learner feels while interacting with the tool, such as curiosity, enthusiasm, excitement, confidence, enjoyment, and a sense of engagement. Feelings of this kind can nudge a student to treat ChatGPT less like a vending machine for answers and more like a learning partner, a source of feedback, and a form of academic support. A student in that frame of mind is also more willing to pose exploratory questions, weigh one explanation against another, go back and revise, and lean on the output to understand something more fully. Why this matters is that academic skill rarely arrives in a single step; it is built up through repeated practice, the reading of feedback, self-correction, and the slow refinement of competence. Empirical studies on AI-supported learning provide support for this argument. Begmatova and Saydazimova (2026) found that AI-assisted instruction improved several academic writing features, including semantic complexity, referential cohesion, lexical diversity, and argumentation. Bauer et al. (2026) reported that ChatGPT improved writing quality and writing experience, with benefits observed during AI use and after students returned to writing without AI assistance. Ayanwale and Omeh (2026) showed that AI-supported problem-based learning improved computational thinking and academic achievement in STEM education. Zheng et al. (2026) found that generative AI literacy was positively associated with higher-order thinking skills both directly and indirectly through learning engagement. These studies indicate that AI can support skill development when students engage with it as an active learning resource. Positive affect may intensify this process by increasing students’ willingness to persist and experiment. For example, curiosity may promote exploration of alternative explanations. Enthusiasm may increase the perceived value of learning interactions. Confidence may encourage students to apply AI-generated suggestions to writing, research, and analysis tasks. Enjoyment may increase sustained engagement. Therefore, positive affect is expected to be positively associated with perceived academic skill development.

### 2.4. Positive Affect and Attitudes Toward ChatGPT

Positive affect is also expected to shape students’ attitudes toward ChatGPT. Emotional experiences during technology use often become evaluative signals. When students experience ChatGPT interactions as enjoyable, confidence-building, stimulating, and useful, they are more likely to develop favourable attitudes toward the tool. Positive affect helps transform immediate emotional experience into a more stable evaluative orientation. Evidence from recent generative AI studies supports this pathway. Q. Q. Chen et al. (2026a) found that generative AI tools enhanced students’ positive affective engagement in source-based academic writing, including inspiration, alertness, attentiveness, and determination. Zhao and Wang (2026) showed that AI feedback improved writing confidence and outcomes, and that improvements in writing confidence were associated with learners’ attitudes toward AI. Ding et al. (2026) found that public evaluations of ChatGPT are shaped by direct encounters with utility, efficiency, reliability concerns, and error experiences, indicating that attitudes toward ChatGPT are grounded in lived interactions rather than abstract assumptions. Kailin and Saeed (2026) further showed that students’ engagement with ChatGPT feedback involved affective responses such as appreciation, curiosity, doubt, and frustration, suggesting that emotional responses form part of the process through which students evaluate AI feedback. In higher education, this kind of affective appraisal carries extra weight, since using ChatGPT so often means working under uncertainty. Students must decide whether responses are reliable, whether outputs are academically appropriate, and whether AI support helps them learn. Positive affect may reduce perceived friction and strengthen favourable interpretations of the tool. Accordingly, students who experience positive emotional states during ChatGPT use are expected to develop more positive attitudes toward ChatGPT.

**H2.**  *Positive affect during ChatGPT use is positively associated with attitudes toward ChatGPT.*

### 2.5. Attitudes Toward ChatGPT and Academic Skill Development

Attitudes toward ChatGPT are expected to be positively associated with perceived academic skill development. Students who believe ChatGPT is useful, effective, and beneficial are more likely to engage with it intentionally and to invest in AI-supported academic tasks. Favourable attitudes may encourage students to use ChatGPT for research planning, writing refinement, feedback interpretation, argument development, problem solving, and conceptual clarification. These activities are closely aligned with the development of academic skills. This relationship is supported by studies showing that positive evaluations of ChatGPT are associated with educational use and learning outcomes. El-Sobkey et al. (2026) reported positive associations between perceived usefulness, ease of use, academic characteristics, and ChatGPT usage patterns in physical therapy education. Ali et al. (2026) found that attitudes toward ChatGPT significantly influenced intentions to use ChatGPT among library and information science students. Alotaibi (2026) showed that AI adoption intentions, which are partly shaped by attitudes, are positively related to students’ critical thinking engagement. Webb et al. (2026) further showed that complementary generative AI use was positively associated with academic performance in English medium instruction, while substitutive use was less beneficial. These findings suggest that favourable attitudes matter most when they support complementary, reflective, and skill-oriented use. Attitude may therefore function as the dominant evaluative driver of academic skill development. Students who view ChatGPT as a legitimate and useful learning tool may be better positioned to use it critically and iteratively. They may also be more likely to maintain engagement long enough for skill gains to emerge. Therefore, attitudes toward ChatGPT are expected to positively predict academic skill development.

**H3.**  *Attitudes toward ChatGPT are positively associated with perceived academic skill development.*

### 2.6. The Mediating Role of Attitude

The mediation pathway from positive affect to attitude and then to academic skill development integrates Broaden-and-Build Theory with the Technology Acceptance Model. Positive affect may broaden students’ engagement with ChatGPT and make AI interactions more exploratory and rewarding. Over time, these positive experiences may become crystallised into favourable attitudes toward ChatGPT. These attitudes may then support sustained, purposeful, and skill-oriented engagement with AI and, in turn, perceived academic skill development. This mediating logic is consistent with recent empirical work. Studies of generative AI adoption show that attitude is an important mechanism connecting students’ perceptions and experiences to behavioural intentions and actual use (Rizun et al., 2026; Alotaibi, 2026; Ali et al., 2026). Studies of AI-supported writing further indicate that students’ emotional and confidence-related experiences are associated with their attitudes toward AI and their writing outcomes (Zhao & Wang, 2026; Q. Q. Chen et al., 2026a). Human–AI collaboration research also shows that students’ critical and metacognitive engagement with ChatGPT can support writing development when AI use is scaffolded and students retain authorial agency (Alyasin & Shah, 2026). In the present model, attitude is therefore expected to explain part of the relationship between positive affect and perceived academic skill development. Positive affect should still have a direct effect because emotions can immediately influence engagement, persistence, and exploratory behaviour. However, part of its effect should operate through attitude, because favourable emotional experiences make students more likely to evaluate ChatGPT positively and use it productively for skill development.

**H4.**  *Attitudes toward ChatGPT mediate the relationship between positive affect and perceived academic skill development.*

### 2.7. Learning Orientation and Desirable Difficulty in AI-Supported Learning

Learning orientation reflects students’ intrinsic motivation to develop competence, master difficult tasks, improve academic skills, and treat challenges as part of the learning process. A student with high learning orientation is more likely to reach for ChatGPT as a way to understand something than as a shortcut around an assignment. Such a student might use the tool to pressure test their reasoning, tighten a draft, work through a concept that is not yet clear, or set rival explanations side by side, all while keeping ownership of the learning. Desirable difficulty theory offers a way to read how this orientation conditions the affective, evaluative, and skill pathways at once (Bjork & Bjork, 2011): deeper learning, it argues, tends to show up when a task carries a manageable degree of challenge rather than when help is frictionless. Carried into ChatGPT-mediated learning, this suggests that a strongly learning-oriented student may not need much positive affect or a particularly favourable attitude to build skill. Their drive toward mastery can already carry them through the uncertainty, the imperfect outputs, and the cognitively demanding back-and-forth. A student with weaker learning orientation, by contrast, is likelier to lean on positive affect and a favourable attitude as scaffolding for staying engaged. Recent work in AI education is consistent with this reading. Urhahne et al. (2026) showed that students’ epistemic beliefs predict ChatGPT adoption and avoidance, suggesting that deeper beliefs about knowledge shape AI engagement. Zheng et al. (2026) found that generative AI literacy profiles differ in learning engagement and higher-order thinking skills, highlighting the role of learner characteristics in AI-supported skill development. Q. Q. Chen et al. (2026a) showed that a growth mindset can reduce the negative effects of perceived skill threat in generative AI integration, indicating that mastery-oriented beliefs can buffer learners against AI-related uncertainty. Meishar Tal and Amzalag (2026) further cautioned that ChatGPT can increase students’ perceived self-efficacy during use while reducing confidence in performing tasks without it, reinforcing the need for learning orientations that preserve independent skill development. Learning orientation is therefore expected to moderate the effects of positive affect and attitude on academic skill development. Specifically, the relationships are expected to weaken at higher levels of learning orientation because intrinsically learning-oriented students can derive skill benefits through effortful engagement and cognitive challenges even when affective or evaluative support is less strong. This does not mean that positive affect and attitude are unimportant. Rather, it suggests that they are more necessary for students who require greater motivational and evaluative scaffolding.

**H5.**  *Learning orientation negatively moderates the relationship between attitudes toward ChatGPT and perceived academic skill development, such that the relationship is weaker at higher levels of learning orientation.*

**H6.**  *Learning orientation negatively moderates the relationship between positive affect and perceived academic skill development, such that the relationship is weaker at higher levels of learning orientation*.

### 2.8. State of the Art in Positive Affect, ChatGPT Use, and Academic Skill Development

Research on ChatGPT in higher education has expanded from general adoption studies to more specialised work on academic writing, affective engagement, learning outcomes, higher-order thinking, ethical use, and learner agency. However, few studies integrate positive affect, attitude, learning orientation, and academic skill development into a single mediated and moderated model. The positioning of the present study within the literature is summarised in Table 1.

Read together, the studies summarised in Table 1 point to a common gap rather than to several separate ones. Studies of adoption and technology acceptance establish that evaluative judgements shape students’ ChatGPT uptake, but they rarely treat the emotional tone of the interactions as a starting point for that evaluation. Work on affective engagement shows that ChatGPT use is emotionally coloured, but it stops short of tracing those emotions through to broad academic skill outcomes. The writing- and skill-focused literature demonstrates that ChatGPT can improve specific competencies, yet it typically examines a single skill domain in isolation rather than a general construct of academic development. Research on higher-order thinking and on learner agency, in turn, shows that engagement quality and motivational stance matter for what students take away from AI-supported learning, without formally testing learning orientation as a moderator of affective or evaluative pathways. No single study in the literature offers an account of how students’ momentary emotional experiences of ChatGPT become translated, through evaluation and subject to motivational boundary conditions, into a general sense of academic growth. That combined gap, rather than any one limitation in isolation, is what the present study’s affective–evaluative moderated framework is designed to close.

### 2.9. Conceptual Framework

**Figure 1:**
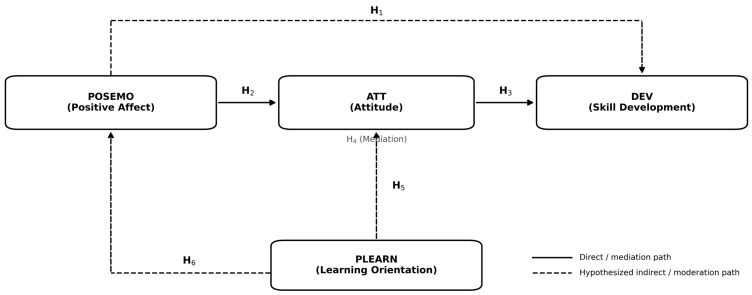
Conceptual model.

The conceptual framework links positive affect, attitudes toward ChatGPT, learning orientation, and academic skill development. Positive affect is expected to directly improve academic skill development and to indirectly improve it through attitudes toward ChatGPT. Attitude is expected to serve as the main evaluative mechanism because students who view ChatGPT favourably are more likely to use it constructively for research, writing, analytical, and field-specific learning tasks. Learning orientation is expected to moderate both the positive affect to academic skill development pathway and the attitude to academic skill development pathway. Specifically, these effects are expected to be weaker when learning orientation is high because learning-oriented students can benefit from cognitive challenges and self-directed engagement without depending as strongly on affective or evaluative scaffolding. Figure 1: Conceptual model of positive affect, attitude, learning orientation, and academic skill development. POSEMO = positive affect; ATT = attitude toward ChatGPT; PLEARN = learning orientation; and DEV = academic skill development. The model specifies direct effects from POSEMO to DEV and ATT, a direct effect from ATT to DEV, an indirect effect from POSEMO through ATT to DEV, and the moderating effects of PLEARN on the ATT to DEV and POSEMO to DEV relationships.

## 3. Materials and Methods

### 3.1. Research Design

This study adopted a quantitative, cross-sectional explanatory research design to examine how positive affect during ChatGPT use predicts academic skill development among higher education students. The design was appropriate because the study sought to test theoretically specified relationships among latent constructs rather than merely describe students’ use of ChatGPT. Specifically, the study examined whether positive affect influences academic skill development directly or indirectly through attitudes toward ChatGPT, and whether learning orientation moderates the affective and evaluative pathways leading to skill development. The analytical model was grounded in an affective–evaluative framework of ChatGPT-mediated learning. In that framework, positive affect plays the upstream emotional role, attitude toward ChatGPT carries the evaluative mediation, learning orientation acts as the moderating condition, and academic skill development is the outcome of interest. A cross-sectional design allowed the study to estimate these links across a large international pool of active ChatGPT users. Such data cannot establish causation, but they are a sound basis for testing the associations, the mediation, and the moderation patterns that sit at the heart of students’ experiences of ChatGPT-supported academic development.

### 3.2. Data Source and Data Collection

The study used data from the Global ChatGPT Student Survey coordinated by CovidSocLab at the University of Ljubljana (Dumlao, 2024; Ravšelj et al., 2025). This global dataset has also been used in other large-scale higher education research on generative AI (Bencsik et al., 2026). The survey was administered online and collected information on students’ use of ChatGPT, emotional experiences during ChatGPT interactions, attitudes toward the tool, learning-related orientations, and perceived academic outcomes. The dataset was suitable for the present study because it includes students from diverse national, institutional, disciplinary, and academic backgrounds, allowing the proposed model to be examined across a broad international higher education context. The survey included items that measured the four central constructs in this study. Positive affect was measured using five items capturing favourable emotional experiences during ChatGPT interactions, including enthusiasm, curiosity, excitement, engagement, and positive valence. Academic skill development was measured using eight items capturing students’ perceived development of research skills, critical thinking, writing, analysis, and field-specific competencies. Attitudes toward ChatGPT were measured through items reflecting perceived usefulness, effectiveness, and students’ general evaluative orientation toward the tool. Learning orientation was measured using items capturing deep learning orientation, mastery motivation, and the use of academic tasks as opportunities for skill development. All items were measured on five-point Likert-type scales ranging from 1, strongly disagree, to 5, strongly agree. The use of Likert-type indicators was appropriate because the model estimated latent constructs that represent students’ affective, evaluative, motivational, and developmental experiences of ChatGPT use. Participation in the original survey was voluntary, and informed consent was obtained before data collection. The analysis was conducted using anonymised secondary data and reported only aggregate results.

To avoid ambiguity about the data source, we clarify the dataset’s exact identity and provenance here. The analysis draws on the early perceptions wave of the global survey on higher education students’ perceptions of ChatGPT, coordinated internationally by CovidSocLab at the University of Ljubljana and administered between October 2023 and February 2024 (Ravšelj et al., 2025). The underlying survey microdata are deposited on Mendeley Data as “Higher Education Students’ Early Perceptions of ChatGPT: Global Survey Data” (DOI:10.17632/ymg9nsn6kn.2), with a companion validation study published in PLoS ONE (https://doi.org/10.1371/journal.pone.0315011). For the present analysis, we used a publicly reposted, restructured version of this same survey, curated on Kaggle as “Global Student Perceptions of ChatGPT” (Dumlao, 2024; https://www.kaggle.com/datasets/jocelyndumlao/global-student-perceptions-of-chatgpt, accessed on 30 April 2026). This is an existing, publicly available secondary dataset rather than an independently collected or adapted sample: no items, cases, or variables were added beyond what appears in the public repository, and no primary data collection was undertaken by the present authors. The dataset was accessed directly from the Kaggle repository cited above and in the Data Availability Statement; the corresponding author holds the exact access date and download record for verification during the review process.

### 3.3. Population and Sample

The target population consisted of undergraduate, postgraduate, and doctoral students who had used ChatGPT for academic purposes. Since the focus was squarely on ChatGPT-mediated skill development, eligibility was limited to active users. The sampling was purposive in that sense: from the global survey pool we kept respondents who reported using ChatGPT and who had answered every indicator in the measurement and structural models. That left 12,035 students for analysis, drawn from 135 countries, which gives the affective–evaluative model a genuinely international footing. The resulting sample size sits well above the usual minimum for partial least squares structural equation modelling, whether one counts indicators, structural paths, mediation, or moderation, and it gave the analysis ample power to pick up the interaction effects tied to learning orientation. The respondents’ demographic profiles are set out in Table 2.

The recruitment strategy for these 12,035 responses proceeded in two sequential stages. At the source survey stage, participants were recruited through a large-scale, multi-country convenience sampling exercise: partner researchers and higher education institutions in the coordinating CovidSocLab network promoted the online questionnaire in classrooms and through university communication channels in each participating country, so that no single national probability frame was used and country-level sample sizes were not proportionate to national student populations. At this study stage, we applied non-probability purposive filtering to this convenience sample, retaining only respondents who reported active ChatGPT use and who had complete responses on every indicator of positive affect, attitude toward ChatGPT, learning orientation, and academic skill development, as described in Section 3.5. Because both stages relied on convenience and purposive procedures rather than on random or stratified sampling, the resulting sample offers broad international coverage but should not be treated as statistically representative of the global higher education student population, a point we return to in the section “Limitations and Future Research Directions”.

Across the profiles, the sample spanned a range of ages, study levels, disciplines, and degrees of AI familiarity. Undergraduates were the largest contingent at 81.8%. The gender split was close to even, with women at 52.1% and men at 47.9%. By discipline, Social Sciences led at 39.4%, with Applied Sciences next at 35.5%. Familiarity with AI varied widely: at one end, 24.9% described themselves as expert; at the other, 15.5% reported no familiarity at all. Regarding income context, two-thirds of respondents (66.0%) came from lower-to-middle-income countries and the remaining 34.0% from high-income ones. Between them, these figures give the analysis a broad international base for studying ChatGPT-mediated academic skill development.

### 3.4. Construct Operationalisation

Four latent constructs formed the core of the model: positive affect, attitude toward ChatGPT, learning orientation, and academic skill development. Positive affect was specified as the independent variable and captured favourable emotional states experienced during ChatGPT use. These emotions reflect the affective quality of AI interactions and are theoretically linked to exploratory engagement, persistence, and broader cognitive processing. Attitude toward ChatGPT was specified as the mediator. This construct captured students’ evaluative orientation toward ChatGPT as a useful, effective, and beneficial academic tool. The mediating role of attitude was included to test whether positive emotional experiences become translated into academic skill development through favourable evaluations of ChatGPT. Learning orientation was specified as the moderator. This construct captured students’ tendencies to approach academic tasks through mastery motivation, deep learning, and competence development. Learning orientation was included to test whether the strength of the positive affect to skill development pathway and the attitude to skill development pathway differs according to students’ motivational orientation. Academic skill development was specified as the dependent variable. This construct captured students’ perceived improvements in research, writing, analytical, critical thinking, and field-specific academic competencies as a result of ChatGPT use. Because this construct is measured entirely through students’ perceptions, it denotes perceived academic skill development, and the label academic skill development is used in these terms throughout. Table 3 summarises the operationalisation of the constructs.

Academic skill development was deliberately specified as a single reflective latent construct rather than as a set of separate skill dimensions, and this choice reflects the study’s theoretical focus on perceived, self-directed academic growth rather than on discipline-specific skill taxonomies. Broaden-and-Build Theory (Fredrickson, 2001) treats the resources that positive emotions help build—cognitive, intellectual, and psychological skills—as a general reservoir rather than as independent capacities, and the Technology Acceptance Model likewise models perceived benefit as a unified evaluative judgement rather than as domain-specific outcomes. Consistent with this framing, research, writing, analytical, critical-thinking, and field-specific competencies were conceptualised as correlated manifestations of a single underlying reflective construct, namely the student’s broad sense that ChatGPT-mediated engagement is helping them grow academically, rather than as distinct constructs that would each require their own antecedents, hypotheses, and structural paths. Modelling these five skill domains as separate sub-constructs would have required a higher-order or multidimensional specification with its own discriminant validity and cross-loading requirements, which was beyond the scope of a study whose central contribution is the affective–evaluative pathway to skill development rather than a taxonomy of skill types.

This unidimensional, reflective specification is also supported empirically. All eight academic skill development indicators loaded on a single factor, with outer loadings between 0.740 and 0.860, well above the 0.60 minimum for reflective indicators. The construct achieved a Cronbach’s alpha of 0.922, a composite reliability of 0.937, and an average variance extracted (AVE) of 0.650, indicating that the eight items share substantially more variance with one another than with error, which would not be expected if the underlying skill domains were empirically distinct. Discriminant validity analysis using the HTMT ratio and the Fornell–Larcker criterion further confirmed that academic skill development was empirically separable from the other latent constructs in the model. Taken together, the consistently high loadings and strong AVE support the treatment of academic skill development as one coherent reflective construct for the purposes of this model; the section “Limitations and Future Research Directions” acknowledges this as a modelling choice with limitations that future research could usefully revisit.

### 3.5. Data Screening and Inclusion Criteria

The data were screened before model estimation. The first inclusion criterion was active ChatGPT use, because the focal constructs, especially positive affect during ChatGPT interactions and academic skill development from ChatGPT, were meaningful only for students who had used the tool. Respondents who had not used ChatGPT were therefore excluded from the analytical sample. The second inclusion criterion was complete data on all selected model indicators. Cases with missing responses on the indicators for positive affect, attitude toward ChatGPT, learning orientation, or academic skill development were excluded from the primary analysis. This complete-case approach ensured that all measurement and structural model estimates were based on the same set of respondents. After screening, the final analytical sample consisted of 12,035 active ChatGPT users. The retained sample was appropriate for PLS-SEM because it exceeded the minimum sample size required for the number of constructs, indicators, and structural paths in the model. It was also suitable for testing mediation and moderation because interaction effects typically require larger samples to achieve adequate statistical power. The large sample therefore strengthened the reliability of the parameter estimates and supported robust hypothesis testing.

Beyond the two inclusion criteria described above, several further steps safeguarded data quality. The source survey embedded attention check items and logical consistency checks during data collection, and the survey administrators removed straight-lining, speeded, and duplicate submissions (for example, repeated submissions from the same IP address or device fingerprint within a short time window) prior to public release. Because this study used the publicly released, already-cleaned file rather than the raw response log, we relied on the administrators’ documented screening and could not independently re-run attention check or duplicate detection routines on discarded cases. On our end, the preprocessing pipeline consisted of the following steps, applied in sequence: (1) importing the publicly released file into statistical software; (2) recoding and reverse-scoring items as required by the original instrument documentation; (3) applying the active-ChatGPT-use filter described above; (4) applying listwise (complete-case) deletion on the positive affect, attitude, learning orientation, and academic skill development indicators; (5) checking the retained cases for out-of-range values and multivariate outliers using Mahalanobis distance, with no cases removed on this basis; and (6) exporting the resulting 12,035-respondent analytical file for measurement and structural model estimation. We report this pipeline in full so that the data preparation steps can be independently reproduced from the public dataset.

### 3.6. Analytical Approach

Partial least squares structural equation modelling was used to estimate the measurement and structural models. PLS-SEM was appropriate for this study for four main reasons. First, the study involved multiple latent variables measured through several observed indicators. Second, the aim of the analysis was explanatory and prediction-oriented, with academic skill development as the main endogenous outcome. Third, the model included both mediation and moderation pathways, which required the simultaneous estimation of direct, indirect, and interaction effects. Fourth, PLS-SEM is suitable for large and complex survey datasets and does not require the same distributional assumptions as covariance-based structural equation modelling. All constructs were specified as reflective measurement models. This specification was appropriate because the observed indicators were treated as manifestations of their underlying latent constructs. For example, the positive affect items were treated as reflective indicators of students’ emotional experiences during ChatGPT use, while the academic skill development items were treated as reflective manifestations of perceived academic skill development. The structural model estimated direct effects from positive affect to academic skill development and from positive affect to attitude toward ChatGPT. It also estimated the direct effect of attitude toward ChatGPT on academic skill development. The mediation analysis tested whether attitude toward ChatGPT transmitted the effect of positive affect to academic skill development. The moderation analysis tested whether learning orientation moderated the relationship between attitude toward ChatGPT and academic skill development, and whether learning orientation moderated the relationship between positive affect and academic skill development. Bootstrapping with 5000 resamples was used to obtain standard errors, t-statistics, *p*-values, and confidence intervals for the hypothesised direct, indirect, and interaction effects. The hypothesis testing procedure therefore allowed the study to evaluate the statistical significance and direction of all six proposed hypotheses.

### 3.7. Measurement Model Assessment

The measurement model was assessed before interpreting the structural paths. Indicator reliability was evaluated using outer loadings. Internal consistency reliability was assessed using Cronbach’s alpha and composite reliability. Convergent validity was assessed using the average variance extracted. Discriminant validity was evaluated using the heterotrait–monotrait ratio and the Fornell–Larcker criterion. These procedures ensured that the constructs were measured reliably and that each construct was empirically distinct from the others. Model fit and explanatory power were also assessed. The standardised root mean square residual was used as a model fit indicator, while the coefficient of determination was used to evaluate the explanatory power of the model for endogenous constructs. Particular attention was given to the R^2^ value for academic skill development because it was the main outcome construct in the study.

### 3.8. Ethical Considerations

The study used anonymised secondary survey data from an international research initiative. The analysis was conducted at the aggregate level and did not involve the identification of individual participants. Informed consent was obtained during the original data collection process. Because what was under study was students’ lived experience of ChatGPT, the findings were read carefully and reported responsibly, with no causal claims pressed beyond what a cross-sectional design can bear. The breadth of the sample called for some sensitivity as well: how students experience ChatGPT is likely to vary from one country, institution, discipline, or level of AI familiarity to the next. The results are therefore best read as large-scale evidence of general tendencies in positive affect, attitude, learning orientation, and academic skill development among active users, with the caveat that local conditions will shape how any given student uses and benefits from generative AI.

## 4. Results

### 4.1. Survey Response Details

Before turning to the model itself, it is worth setting the analytical sample in context. In total, 12,035 active ChatGPT-using students from 135 countries took part. Men and women were fairly evenly represented: 5760 men (47.9%) and 6275 women (52.1%). Undergraduates dominated at 81.8%, with postgraduates at 12.2% and doctoral students at 6.0%. Among disciplines, Social Sciences came first with 4739 respondents (39.4%), and Applied Sciences followed at 4276 (35.5%). Natural Sciences and Arts and Humanities accounted for 10.7% and 14.4%, respectively. Reported AI familiarity ran the full range, from 24.9% at expert level and 19.3% very familiar, to 16.4% moderately familiar and 23.9% with a basic understanding, down to 15.5% with none at all. In terms of income context, 66.0% came from lower-to-middle-income countries and 34.0% from high-income ones, a spread that gives the study a wide international footing for examining ChatGPT-enhanced academic skill development across varied higher education settings.

### 4.2. Descriptive Statistics

The descriptive statistics for the study variables are presented in Table 4. All variables were measured on five-point Likert-type scales, with higher scores indicating stronger endorsement of the relevant construct. The mean score for positive affect was 3.018, indicating a moderate level of favourable emotional engagement during ChatGPT use. Attitude toward ChatGPT had a mean of 3.582, suggesting that respondents generally held a favourable evaluative orientation toward ChatGPT. Academic skill development had a mean of 3.400, indicating that students reported meaningful skill-related benefits from ChatGPT use.

Learning orientation had a mean of 3.443, reflecting a moderate-to-high orientation toward mastery, deep learning, and academic skill development. Satisfaction and ChatGPT use intensity were also included in the descriptive and measurement assessments because they formed part of the broader construct set in the dataset. Satisfaction had a mean of 3.343, while ChatGPT use intensity had a lower mean of 2.323. This indicates that students evaluated ChatGPT relatively positively, but their intensity of use varied and remained more moderate. Skewness across the variables fell between −0.461 and 0.595, and kurtosis between −0.438 and 0.498. These values indicate acceptable distributional properties for the variables and support the suitability of the dataset for PLS-SEM estimation.

### 4.3. Reliability and Convergent Validity

The reliability and convergent validity results are presented in Table 5. Internal consistency reliability was assessed using Cronbach’s alpha, composite reliability rho_a, and composite reliability rho_c. The results show that all constructs achieved acceptable reliability values. Cronbach’s alpha ranged from 0.749 for attitude toward ChatGPT to 0.922 for academic skill development. Although the alpha value for attitude was slightly below 0.75, it remained above the commonly accepted threshold of 0.70, indicating acceptable internal consistency.

The remaining constructs exceeded 0.75, confirming strong reliability. Composite reliability rho_c ranged from 0.848 to 0.937, exceeding the recommended threshold of 0.70 for all constructs. The average variance extracted values ranged from 0.528 to 0.779, with all values above the recommended threshold of 0.50. These findings confirm convergent validity, indicating that each construct explained more than half of the variance in its indicators. The measurement model therefore demonstrated adequate reliability and convergent validity.

### 4.4. Discriminant Validity

Discriminant validity was assessed using the heterotrait–monotrait ratio and the Fornell–Larcker criterion. The HTMT results are presented in Table 6. All HTMT values were below the conservative threshold of 0.85. The highest HTMT value was 0.762, between attitude toward ChatGPT and academic skill development, which remained within the acceptable range. This indicates that the constructs were empirically distinct and that no problematic overlap was detected among the latent variables.

The Fornell–Larcker results are presented in Table 7. The square root of AVE for each construct, reported on the diagonal, exceeded the correlations with all other constructs.

For example, the diagonal value for academic skill development was 0.806, which exceeded its correlations with positive affect, attitude, satisfaction, use intensity, and learning orientation. Similarly, the diagonal values for positive affect, attitude, satisfaction, use intensity, and learning orientation exceeded their respective off-diagonal correlations. These results further confirm discriminant validity.

### 4.5. Indicator Loadings

The indicator loadings are presented in Table 8. The loadings for positive affect ranged from 0.614 to 0.855. Although the first positive affect item had a lower loading of 0.614, it remained above the minimum acceptable threshold of 0.60 and was retained because the construct-level reliability and AVE values were acceptable. The attitude toward ChatGPT indicators ranged from 0.792 to 0.853, indicating strong item alignment with the latent construct. Satisfaction indicators ranged from 0.862 to 0.919, confirming strong measurement quality.

The ChatGPT use intensity indicators ranged from 0.696 to 0.754, while learning orientation indicators ranged from 0.839 to 0.908. Academic skill development indicators ranged from 0.740 to 0.860. These results confirm that all indicators loaded adequately on their intended constructs. The measurement model therefore provided a reliable empirical foundation for estimating the structural relationships.

### 4.6. Model Fit

The model fit indices are reported in Table 9. On the main indicators, the estimated model cleared the recommended thresholds. The ratio of chi-square to degrees of freedom came to 2.893, under the customary ceiling of 3, and the RMSEA was 0.071, inside the 0.08 cut-off.

GFI reached 0.918, NFI 0.921, TLI 0.986, and CFI 0.919, each clearing the 0.90 mark, while the SRMR stood at 0.062, comfortably below the 0.10 maximum. Taken together, the model fits the data acceptably and was a sound basis for the structural analysis that follows.

### 4.7. Regression Analysis

As a first step, and before fitting the full structural model, we ran a simple regression to gauge the baseline link between positive affect and academic skill development; Table 10 reports the result. The model produced an R value of 0.519, indicating a moderate positive association between positive affect and academic skill development. The R^2^ value was 0.269, showing that positive affect alone explained 26.9% of the variance in academic skill development. The adjusted R^2^ value was also 0.269, confirming that the explanatory value of the model was stable. The Durbin–Watson value was 1.947, indicating no evidence of problematic autocorrelation in the residuals.

The ANOVA results are presented in Table 11. The regression model was statistically significant: F(1, 12,033) = 4428.750, *p* < 0.001. The regression sum of squares was 3237.809, while the residual sum of squares was 8797.191.

These results confirm that positive affect is a statistically significant predictor of academic skill development when examined as a single predictor. This finding provides preliminary support for the affective pathway proposed in the study before the full PLS-SEM model is interpreted.

### 4.8. Structural Model

**Figure 2:**
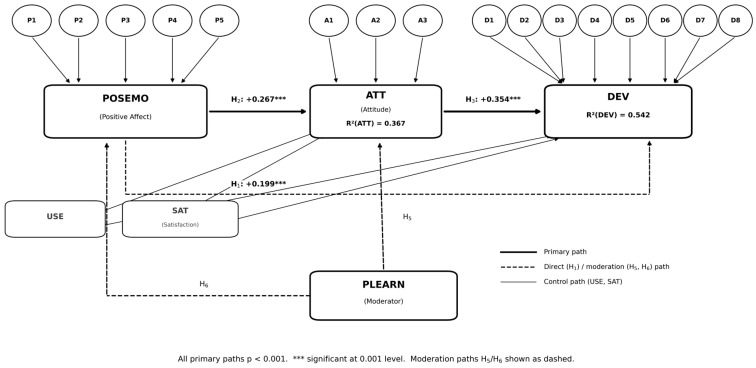
Structural model of positive affect, attitude, learning orientation, and academic skill development.

The structural model was estimated using PLS-SEM with bootstrapping based on 200 resamples. The model examined the direct effect of positive affect on academic skill development, the effect of positive affect on attitude toward ChatGPT, the effect of attitude toward ChatGPT on academic skill development, the mediating role of attitude, and the moderating role of learning orientation. The structural model included positive affect, attitude toward ChatGPT, academic skill development, and learning orientation as the focal constructs. The model explained a substantial proportion of variance in academic skill development, with R^2^(DEV) = 0.542. In other words, positive affect, attitude toward ChatGPT, learning orientation, and the interaction terms together accounted for 54.2% of the variation in students’ perceived academic skill development from using ChatGPT. For the mediator, R^2^(ATT) = 0.367, so positive affect on its own explained 36.7% of the variance in attitude toward ChatGPT. These values demonstrate strong explanatory power for the main endogenous outcome and moderate explanatory power for the mediating construct. Figure 2 presents the structural model. All primary hypothesised paths were statistically significant at *p* < 0.001. The model therefore provides empirical support for the proposed affective–evaluative framework of ChatGPT-mediated academic skill development. The path coefficients, mediation effects, and moderation effects are reported in the following hypothesis testing section.

All primary paths are significant at *p* < 0.001. R^2^(DEV) = 0.542; R^2^(ATT) = 0.367. Dashed lines represent moderation paths involving learning orientation. POSEMO = positive affect; ATT = attitude toward ChatGPT; DEV = academic skill development; and PLEARN = learning orientation.

## 5. Hypothesis Testing

### 5.1. Direct Path Coefficients

The structural path results were examined to test the direct hypotheses in the proposed affective–evaluative model. The results are presented in Table 12. The findings show that all three direct hypotheses were statistically supported at *p* < 0.001. Positive affect was positively and significantly associated with academic skill development (β = 0.199, t = 27.455, *p* < 0.001), supporting H1. This result indicates that students who experienced stronger positive affect during ChatGPT use reported higher levels of academic skill development.

The finding confirms that favourable emotional engagement with ChatGPT is directly associated with perceived improvements in research, writing, analytical, critical thinking, and field-specific academic competencies. Positive affect was also positively and significantly associated with attitude toward ChatGPT (β = 0.267, t = 31.763, *p* < 0.001), supporting H2. This result shows that students who experienced stronger positive emotions during ChatGPT interactions were more likely to develop favourable evaluations of the tool. Positive affect therefore appears to shape students’ evaluative orientation toward ChatGPT, confirming the theoretical assumption that emotional experience during AI use is associated with positive attitudes toward the technology. Attitude toward ChatGPT was positively and significantly associated with academic skill development (β = 0.354, t = 45.314, *p* < 0.001), supporting H3. This was the strongest direct path in the model, indicating that attitude was the dominant direct predictor of perceived academic skill development. Students who evaluated ChatGPT as useful, effective, and beneficial were more likely to report stronger academic skill gains. This finding supports the Technology Acceptance Model logic that favourable evaluative orientations toward technology are important predictors of productive learning-related outcomes.

### 5.2. Mediating and Moderating Effects

The mediation and moderation results are presented in Table 13. The indirect pathway from positive affect to academic skill development through attitude toward ChatGPT was positive and statistically significant (β = 0.095, t = 24.923, *p* < 0.001), supporting H4. The 95% bootstrap confidence interval [0.087, 0.101] excluded zero, confirming the significance of the indirect effect. This result indicates that attitude partially mediates the relationship between positive affect and perceived academic skill development. Positive affect is therefore associated with perceived academic skill development both directly and indirectly through students’ favourable attitudes toward ChatGPT. The first moderation effect examined whether learning orientation moderated the relationship between attitude toward ChatGPT and academic skill development. The interaction term was negative and statistically significant (β = −0.021, t = 3.831, *p* < 0.001), supporting H5.

What this says is that attitude’s positive pull on academic skill development eases off as learning orientation rises. Put plainly, a strongly learning-oriented student leans less on a favourable view of ChatGPT to make progress, because the drive toward mastery is already carrying them into deeper engagement with the work. The second interaction asked the same of the positive affect pathway. Here too, the term was negative and significant (β = −0.045, t = 6.861, *p* < 0.001), which supports H6: the direct effect of positive affect on skill development is dampened when learning orientation is high. That fits the desirable difficulty reading, in which the most learning-oriented students keep gaining from ChatGPT through effortful, self-directed, cognitively demanding work, even when feeling good about the tool matters less to how they learn.

### 5.3. Interpretation of the Moderation Plots

**Figure 3:**
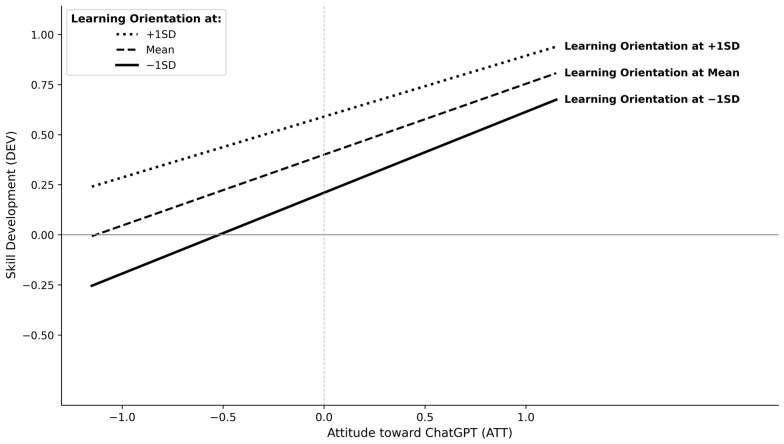
Moderating effect of learning orientation on the relationship between attitude toward ChatGPT and academic skill development.

To read the interactions more closely, we plotted simple slopes. Figure 3 traces how learning orientation conditions the link between attitude toward ChatGPT and academic skill development. The lines stay positive at every level of learning orientation, but they flatten where learning orientation is high. This indicates that favourable attitudes toward ChatGPT are especially important for students with lower learning orientation, who may require stronger evaluative support to link ChatGPT use to perceived academic skill development. For students with higher learning orientation, the attitude pathway remains positive but less steep, suggesting that mastery motivation partly substitutes for attitude-based scaffolding.

**Figure 4:**
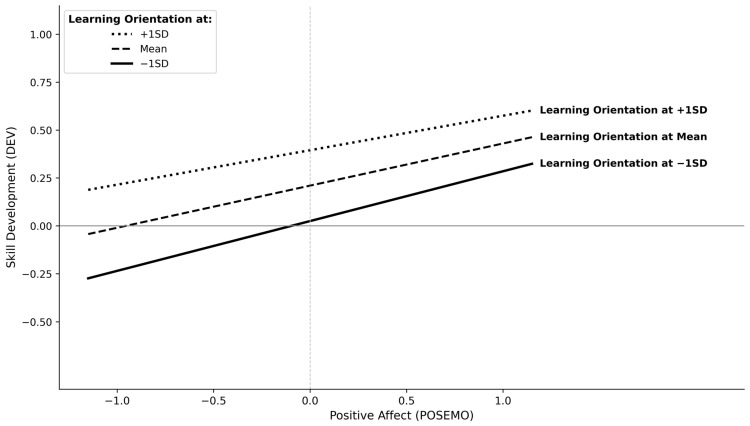
Moderating effect of learning orientation on the relationship between positive affect and academic skill development.

Figure 4 presents the moderating effect of learning orientation on the relationship between positive affect and academic skill development. The slope pattern shows that positive affect has the strongest association with academic skill development when learning orientation is low. As learning orientation increases, the slope becomes weaker.

This indicates that positive affect is most important for students who require stronger affective support to engage productively with ChatGPT. For highly learning-oriented students, academic skill development appears less dependent on positive emotional stimulation because these students are already motivated to engage deeply with academic tasks and tolerate the cognitive demands of AI-supported learning.

### 5.4. Summary of Hypothesis Testing

The hypothesis testing results provide full empirical support for the proposed model. H1 confirmed that positive affect is directly and positively associated with academic skill development. H2 confirmed that positive affect is positively associated with attitude toward ChatGPT. H3 confirmed that attitude toward ChatGPT is the strongest direct predictor of academic skill development. H4 confirmed that attitude partially mediates the relationship between positive affect and academic skill development. H5 and H6 confirmed that learning orientation negatively moderates both the attitude-based and affective pathways to academic skill development. The results show that positive emotional engagement with ChatGPT is associated with perceived academic skill development through both direct and attitude-mediated mechanisms. However, the strength of these pathways depends on students’ learning orientation. The negative moderation effects show that students with stronger learning orientation require less affective and evaluative scaffolding for perceived academic skill development, consistent with the desirable difficulty logic underlying the study.

## 6. Discussion

This study examined how positive affect during ChatGPT use is associated with perceived academic skill development among higher education students, with attitude toward ChatGPT as a mediating mechanism and learning orientation as a moderating condition. On the whole, the data back the affective–evaluative model fairly firmly. Positive affect was a significant predictor of perceived academic skill development (H1: β = 0.199, *p* < 0.001): the students who felt more positive during ChatGPT use also reported building more academic skill. This result supports the Broaden-and-Build logic that favourable emotional states can broaden exploratory engagement, increase persistence, and support the development of durable academic resources. The findings also show that positive affect significantly predicted attitudes toward ChatGPT (H2: β = 0.267, *p* < 0.001). This indicates that students’ emotional experiences during ChatGPT interactions shape their evaluative orientation toward the tool. Students who experience ChatGPT use as stimulating, useful, confidence-building, or engaging are more likely to develop favourable attitudes toward it. In this sense, positive affect is not merely an emotional by-product of AI interactions. It functions as an antecedent of students’ broader evaluation of ChatGPT as an academic support tool.

Attitude toward ChatGPT was the strongest direct predictor of perceived academic skill development (H3: β = 0.354, *p* < 0.001). This finding confirms the central role of evaluative orientation in ChatGPT-mediated learning. Students who hold favourable attitudes toward ChatGPT are more likely to use it purposefully for academic tasks, interpret its outputs constructively, and integrate AI support into research, writing, analytical, and field-specific learning activities. This result is consistent with the Technology Acceptance Model because it shows that positive evaluations of certain technologies are associated with productive academic outcomes. The mediation result further clarifies how positive affect is associated with perceived academic skill development. Attitude partially mediated the relationship between positive affect and academic skill development (H4: β = 0.095, 95% CI = [0.087, 0.101], *p* < 0.001). This indicates that positive emotional experiences with ChatGPT become partly crystallised into favourable attitudes, which then support academic skill gains. The mediation was partial rather than full, meaning that positive affect also retained a direct effect on skill development beyond attitude formation. This suggests that positive emotions relate to learning through both immediate engagement mechanisms and more stable evaluative mechanisms. The most theoretically distinctive finding concerns the moderating role of learning orientation. Learning orientation negatively moderated the relationship between attitude and perceived academic skill development (H5: β = −0.021, *p* < 0.001) and the relationship between positive affect and perceived academic skill development (H6: β = −0.045, *p* < 0.001). These negative interaction effects indicate that the positive associations of attitude and positive affect with perceived academic skill development become weaker when learning orientation is high. This pattern supports a desirable difficulty interpretation. Students with stronger learning orientation may depend less on affective enjoyment or favourable attitudes because their mastery motivation enables them to engage deeply with ChatGPT even when learning requires effort, evaluation, and cognitive challenges. This finding challenges the assumption that stronger positive affect always produces proportionally stronger learning benefits. Positive affect remains beneficial, but its relative importance differs across learners. For students with lower learning orientation, positive affect and favourable attitudes appear to provide important scaffolding for engagement and skill development. For students with higher learning orientation, skill development appears to follow a more self-directed pathway because these students are already motivated to use ChatGPT as a resource for mastery, critical reflection, and academic improvement. Thus, the findings show that ChatGPT-mediated skill development is not associated with affective experience alone. It also depends on the motivational orientation students bring to AI-supported learning. The large international sample of 12,035 students from 135 countries strengthens the relevance of these findings. The results suggest that positive affect, attitude, and learning orientation are important mechanisms in ChatGPT-supported perceived academic skill development across diverse higher education contexts. The model explained the substantial proportion of variance in perceived academic skill development (R^2^ = 0.542), indicating that the affective–evaluative framework has strong explanatory value. The study demonstrates that the academic benefits of ChatGPT depend not only on access or frequency of use, but also on how a student feels while interacting with it, how they judge the tool, and whether they treat learning as a matter of mastery and cognitive challenge.

This desirable difficulty pattern also merits a fuller theoretical explanation, together with its practical implications. Theoretically, the moderation results are consistent with self-determination accounts of intrinsic motivation, in which mastery-oriented learners derive engagement from the inherent challenge and interest in a task rather than from external or affective reinforcement (Bjork & Bjork, 2011). For a highly learning-oriented student, a ChatGPT interaction that feels neutral, effortful, or only moderately enjoyable is still pursued to completion and still mined for learning value, because the motivating force comes from the student’s own mastery goals rather than from how the interaction feels in the moment or how favourably the tool is evaluated. By contrast, for a student with lower learning orientation, continued engagement appears to depend more heavily on the interaction feeling rewarding and on the tool being judged as useful and trustworthy; positive affect and favourable attitude are, in effect, doing motivational work that intrinsic mastery goals would otherwise do. Practically, this implies that affective and evaluative scaffolding, such as encouraging, low-friction onboarding to ChatGPT, prompts that build early confidence, and messaging that frames the tool as trustworthy and useful, is likely to yield the largest returns for students who are not yet strongly learning-oriented. For already mastery-motivated students, institutions may instead gain more by protecting productive difficulty, for instance by pairing ChatGPT use with reflective or evaluative tasks, rather than by further smoothing the emotional experience. A blanket strategy of maximising positive affect and favourable attitudes for all students risks over-investing in scaffolding that highly learning-oriented students do not need, while under-investing in the structured, effortful engagement that would most benefit them.

## 7. Conclusions

What this study shows is that positive affect during ChatGPT use is meaningfully associated with perceived academic skill development, both directly and by way of attitude. Positive affect predicted both skill development and attitude toward ChatGPT, and attitude turned out to be the strongest direct predictor of the two. The mediation analysis confirmed that good emotional experiences crystallise, in part, into positive evaluations of ChatGPT, and that those evaluations then carry through to skill gains. Learning orientation, for its part, moderated both pathways in the negative direction: the more learning-oriented the student, the less they relied on affective or evaluative scaffolding, since the drive toward mastery already sustained a deeper, more self-directed way of working with the tool. The key implication is that AI pedagogy should not focus only on making ChatGPT use enjoyable or easy. It should also cultivate learning orientation, preserve cognitive challenge, and help students use ChatGPT as a tool for developing durable academic skills.

### Limitations and Future Research Directions

This study has several limitations that qualify the findings. First, and most importantly, academic skill development, along with positive affect, attitude toward ChatGPT, and learning orientation, were measured entirely through students’ self-reported perceptions rather than through objective, externally verified performance indicators such as grades, writing rubrics, or task-based skill assessments. Self-reported skill development captures students’ subjective sense of growth, which is a meaningful outcome in its own right, but it need not correspond closely to objectively measured competence gains, and it is vulnerable to social desirability, motivated reasoning, and recall effects. Second, because all constructs were measured using the same survey instrument, at the same point in time, and for the same respondents, the results are exposed to common method variance: shared method effects, such as a general tendency to respond positively across items or consistency motifs in how students answer adjacent questions, could inflate the associations among positive affect, attitude, learning orientation, and academic skill development beyond their true substantive relationships. We did not administer a marker variable or procedurally separate the measurements of predictor and outcome constructs by time or source, which would be the strongest safeguards against this bias. The consistently high indicator loadings and construct reliabilities reported in Section 4 are therefore evidence of measurement quality within this single-method design, not evidence against common method bias as such.

We also want to be explicit about why self-report measures were used, rather than treating this only as a weakness to be noted in passing. In a study spanning 135 countries and a comparably wide range of institutions, disciplines, and grading systems, objective performance indicators such as grades or standardised skill assessments are not comparable across contexts: grading scales, assessment formats, and academic standards differ substantially by country and institution, which would have made a cross-nationally comparable objective measure of academic skill development difficult to construct without a bespoke, resource-intensive assessment protocol administered uniformly worldwide. Self-report measures of perceived academic skill development, by contrast, can be worded consistently and interpreted on a common Likert-type metric regardless of local grading conventions, which is why this approach is the norm in large international survey research on ChatGPT and higher education (e.g., Ravšelj et al., 2025; Dumlao, 2024). This was therefore a deliberate trade-off, offering broad international comparability and a very large, diverse sample at the cost of the objectivity that a single-institution, performance-based design could have offered.

Future research should build on the affective–evaluative model tested here by triangulating self-reported academic skill development with objective indicators, for example course grades, instructor- or rubric-based writing and analytical assessments, or task-based competence tests administered before and after a period of ChatGPT-supported work, ideally within a smaller set of institutions where grading standards are comparable. Longitudinal or experimental designs would also help to establish the direction of the effects proposed here, since the present cross-sectional data can support the associations, mediation, and moderation patterns reported in Section 4 and Section 5, but cannot rule out reverse or reciprocal causation between attitude, affect, and perceived academic skill development. Finally, future studies could usefully test whether the learning orientation moderation reported here (Section 6) is replicated when academic skill development is captured objectively rather than perceptually, which would help clarify whether the desirable difficulty pattern reflects a genuine reduction in the need for affective and evaluative scaffolding, or simply a reduction in how much scaffolding highly learning-oriented students subjectively believe they need.

## Figures and Tables

**Table 1:** Summary of existing work and the present study.

Category	Ref.	Focus	Strengths	Limitations
AI adoption, ethics, and technology acceptance	Rizun et al. (2026); Alotaibi (2026); El-Sobkey et al. (2026); Ali et al. (2026)	Student adoption of ChatGPT, perceived usefulness, ease of use, attitudes, behavioural intentions, ethical concerns, and critical thinking engagement	Establishes attitude as a central evaluative mechanism in AI adoption and educational use	Gives limited attention to positive affect as an upstream emotional predictor of academic skill development
Affective engagement and emotional responses to ChatGPT	Z. Chen et al. (2026b); Zhao and Wang (2026); Kailin and Saeed (2026)	Emotions, affective engagement, confidence, feedback experiences, and learner responses during AI-supported writing	Demonstrates that AI interactions can shape positive affect, confidence, curiosity, doubt, frustration, and engagement	Rarely tests whether positive affect predicts broader academic skill development through attitude
AI-supported writing and academic skill development	Begmatova and Saydazimova (2026); Bauer et al. (2026); Z. Chen et al. (2026b); Papanastasiou et al. (2026)	ChatGPT and AI-assisted academic writing, writing quality, writing confidence, self-efficacy, and skill development	Provides direct evidence that AI can improve writing-related skills and learning experiences	Often focuses on writing outcomes rather than a general academic skill development construct
Higher-order thinking, computational thinking, and critical engagement	Ayanwale and Omeh (2026); Zheng et al. (2026); Sajidin (2026)	Computational thinking, higher-order thinking, critical thinking, engagement, and AI literacy	Shows that AI-supported learning can contribute to complex cognitive skills when students engage actively	Does not fully explain how emotional and evaluative pathways jointly influence skill development
Human–AI collaboration, learner agency, and growth orientation	Alyasin and Shah (2026); Castro-Lopez et al. (2026); Z. Chen et al. (2026b); Urhahne et al. (2026)	Learner agency, metacognition, practical creativity, growth mindset, epistemic beliefs, and engagement with generative AI	Highlights that AI learning benefits depend on learner agency, mindset, epistemic beliefs, and critical engagement	Does not directly test learning orientation as a moderator of affective and evaluative pathways
Present study	Current study	Positive affect as a predictor of academic skill development, attitude as a mediator, and learning orientation as a moderator	Develops an affective–evaluative moderated mediation framework using a large international sample of active ChatGPT users	Cross-sectional design limits causal inference, and self-reported skill development should be complemented by objective measures

**Table 2:** Demographic characteristics of respondents.

Characteristics	Category	Frequency	%
Age	18–24	1841	15.3
	25–34	2210	18.4
	35–44	3003	25.0
	45–54	2978	24.7
	55 and above	2003	16.6
Gender	Male	5760	47.9
	Female	6275	52.1
Educational background	Undergraduate	9847	81.8
	Postgraduate	1472	12.2
	Doctoral	716	6.0
Academic GPA	Below 3.0	1688	14.0
	3.0	5005	41.6
	Above 3.0	5342	44.4
Field of study	Social Sciences	4739	39.4
	Applied Sciences	4276	35.5
	Natural Sciences	1291	10.7
	Arts and Humanities	1729	14.4
AI familiarity	Not familiar at all	1860	15.5
	Basic understanding	2881	23.9
	Moderately familiar	1969	16.4
	Very familiar	2326	19.3
	Expert level	2999	24.9
Country income	High-income countries	4086	34.0
	Lower-to-middle-income countries	7949	66.0
Countries	135 countries		
Total		12,035	100

**Table 3:** Construct operationalisation.

Construct	Abbreviation	Role in Model	Indicators	Conceptual Meaning
Positive affect	POSEMO	Independent variable	Five emotion items	Favourable emotional experiences during ChatGPT use, including enthusiasm, curiosity, excitement, engagement, and positive valence
Attitude toward ChatGPT	ATT	Mediator	Three evaluative items	Students’ evaluative orientation toward ChatGPT as useful, effective, and beneficial for academic purposes
Learning orientation	PLEARN	Moderator	Three learning-orientation items	Students’ mastery motivation, deep learning approach, and orientation toward academic skill development
Academic skill development	DEV	Dependent variable	Eight skill-development items	Perceived development of research, writing, analytical, critical thinking, and field-specific academic competencies

**Table 4:** Descriptive statistics.

Variable	Min	Max	Mean	Std. Dev.	n	Skewness	Kurtosis
Positive affect	1	5	3.018	0.939	12,035	−0.184	−0.438
Attitude toward ChatGPT	1	5	3.582	0.792	12,035	−0.367	0.239
Satisfaction	1	5	3.343	0.828	12,035	−0.391	0.246
ChatGPT use intensity	1	5	2.323	0.855	12,035	0.595	−0.109
Learning orientation	1	5	3.443	0.844	12,035	−0.461	0.471
Academic skill development	1	5	3.400	0.801	12,035	−0.425	0.498

Note: All variables were measured on five-point Likert-type scales, where 1 = strongly disagree and 5 = strongly agree. n = 12,035.

**Table 5:** Reliability and convergent validity.

Construct	Cronbach’s Alpha	Composite Reliability rho_a	Composite Reliability rho_c	AVE
Positive affect	0.830	0.881	0.881	0.601
Attitude toward ChatGPT	0.749	0.857	0.857	0.667
Satisfaction	0.857	0.913	0.913	0.778
ChatGPT use intensity	0.778	0.848	0.848	0.528
Learning orientation	0.856	0.914	0.914	0.779
Academic skill development	0.922	0.937	0.937	0.650

Note: AVE = average variance extracted. AVE values above 0.50 indicate convergent validity. Composite reliability values above 0.70 indicate acceptable reliability.

**Table 6:** Discriminant validity using the HTMT ratio.

Construct	POSEMO	ATT	SAT	USE	PLEARN	DEV
POSEMO						
ATT	0.605					
SAT	0.469	0.603				
USE	0.503	0.513	0.340			
PLEARN	0.412	0.544	0.454	0.370		
DEV	0.592	0.762	0.580	0.482	0.591	

Note: HTMT values below 0.85 indicate acceptable discriminant validity. POSEMO = positive affect; ATT = attitude toward ChatGPT; SAT = satisfaction; USE = ChatGPT use intensity; PLEARN = learning orientation; and DEV = academic skill development.

**Table 7:** Discriminant validity using the Fornell–Larcker criterion.

Construct	(1)	(2)	(3)	(4)	(5)	(6)
(1) POSEMO	0.775					
(2) ATT	0.479	0.817				
(3) SAT	0.397	0.487	0.882			
(4) USE	0.411	0.405	0.285	0.727		
(5) PLEARN	0.347	0.437	0.390	0.308	0.883	
(6) DEV	0.519	0.636	0.518	0.417	0.526	0.806

Note: Diagonal values represent the square root of AVE. Diagonal values should exceed the off-diagonal correlations in the corresponding rows and columns.

**Table 8:** Indicator loadings.

Construct	Item	Outer Loading
Positive affect	POSEMO 1	0.614
	POSEMO 2	0.838
	POSEMO 3	0.757
	POSEMO 4	0.855
	POSEMO 5	0.786
Attitude toward ChatGPT	ATT 1	0.792
	ATT 2	0.853
	ATT 3	0.803
Satisfaction	SAT 1	0.862
	SAT 2	0.919
	SAT 3	0.863
ChatGPT use intensity	USE 1	0.741
	USE 2	0.754
	USE 3	0.696
	USE 4	0.731
	USE 5	0.710
Learning orientation	PLEARN 1	0.839
	PLEARN 2	0.900
	PLEARN 3	0.908
Academic skill development	DEV 1	0.777
	DEV 2	0.775
	DEV 3	0.740
	DEV 4	0.838
	DEV 5	0.826
	DEV 6	0.794
	DEV 7	0.860
	DEV 8	0.832

**Table 9:** Model fit indices.

Index	Recommended Value	Estimated Model
Chi-square/df	<3	2.893
RMSEA	<0.08	0.071
GFI	>0.90	0.918
AGFI	>0.80	0.844
SRMR	<0.10	0.062
NFI	>0.90	0.921
TLI	>0.90	0.986
CFI	>0.90	0.919

**Table 10:** Regression model summary.

Model	R	R^2^	Adjusted R^2^	SE Estimate	R^2^ Change	F Change	df1	df2	Sig. F Change
1	0.519	0.269	0.269	0.8550	0.269	4428.750	1	12,033	0.000

Note: Predictor = positive affect. Dependent variable = academic skill development.

**Table 11:** Regression ANOVA results.

Model	Sum of Squares	df	Mean Square	F	Sig.
Regression	3237.809	1	3237.809	4428.750	0.000
Residual	8797.191	12,033	0.731		
Total	12,035.000	12,034			

Note: Dependent variable = academic skill development. Predictor = positive affect.

**Table 12:** Direct path coefficients.

Hypothesis	Path	Original Sample (O)	Sample Mean (M)	STDEV	t-Statistics	*p*-Values	Decision
H1	Positive affect → Academic skill development	0.199	0.201	0.007	27.455	0.000	Supported
H2	Positive affect → Attitude toward ChatGPT	0.267	0.269	0.008	31.763	0.000	Supported
H3	Attitude toward ChatGPT → Academic skill development	0.354	0.352	0.008	45.314	0.000	Supported

Note: STDEV = bootstrapped standard error. *p* = 0.000 is reported as *p* < 0.001 in the interpretation.

**Table 13:** Mediation and moderation effects.

Hypothesis	Path	Original Sample (O)	Sample Mean (M)	STDEV	t-Statistics	*p*-Values	Decision
H4	Positive affect → Attitude toward ChatGPT → Academic skill development	0.095	0.096	0.004	24.923	0.000	Supported
H5	Learning orientation × Attitude toward ChatGPT → Academic skill development	−0.021	−0.020	0.005	3.831	0.000	Supported
H6	Learning orientation × Positive affect → Academic skill development	−0.045	−0.044	0.007	6.861	0.000	Supported

Note: STDEV = bootstrapped standard error. The 95% bootstrap confidence interval for H4 was [0.087, 0.101]. *p* = 0.000 is reported as *p* < 0.001 in the interpretation.

## Data Availability

This study analyses the publicly available Global Student Perceptions of ChatGPT dataset, accessible through Kaggle at https://www.kaggle.com/datasets/jocelyndumlao/global-student-perceptions-of-chatgpt (accessed on 30 April 2026). Derived analysis files are available from the corresponding author upon reasonable request. To further support transparency and reproducibility, the preprocessing pipeline described in Section 3.5 (recoding, filtering, listwise deletion, and outlier screening), together with the syntax used to reproduce the 12,035-respondent analytical file from the public Kaggle/Mendeley release, are available from the corresponding author upon reasonable request. A separate anonymised extract of the analytical file was not deposited in a new public repository because the source data are already publicly archived in full on Kaggle and Mendeley Data under the licences set by the original data custodians (Dumlao, 2024; Ravšelj et al., 2025); redistributing a derived subset would duplicate that existing public archive rather than add a new one.

## Abbreviations

The following abbreviations are used in this manuscript:

Academic GPA	Respondents’ reported academic grade point average category
Academic Skill Development	Main dependent variable measuring perceived development of research, writing, analytical, critical thinking, and field-specific competencies
AGFI	Adjusted goodness-of-fit index
AI	Artificial intelligence
AI Familiarity	Respondents’ self-reported level of familiarity with artificial intelligence
Applied Sciences	Field of study category in the demographic profile
Arts and Humanities	Field of study category in the demographic profile
ATT	Attitude toward ChatGPT
ATT 1	First indicator measuring attitude toward ChatGPT
ATT 2	Second indicator measuring attitude toward ChatGPT
ATT 3	Third indicator measuring attitude toward ChatGPT
Attitude toward ChatGPT	Mediating construct capturing students’ evaluative orientation toward ChatGPT as useful, effective, and beneficial for academic purposes
AVE	Average variance extracted
β	Standardised path coefficient
Basic understanding	AI familiarity category indicating basic knowledge of artificial intelligence
CFA	Confirmatory factor analysis
CFI	Comparative fit index
ChatGPT	Conversational generative artificial intelligence tool examined in the study
ChatGPT Use Intensity	Construct measuring the extent or frequency of students’ ChatGPT use
Chi-square/df	Chi-square divided by degrees of freedom
CI	Confidence interval
Composite reliability rho_a	Reliability coefficient used to assess construct internal consistency
Composite reliability rho_c	Composite reliability coefficient used to assess construct internal consistency
Country Income	Demographic classification distinguishing high-income and lower-to-middle-income country contexts
Countries	Number of country contexts represented in the sample
CovidSocLab	Research unit at the University of Ljubljana associated with the Global ChatGPT Student Survey
CR	Composite reliability
Cronbach’s alpha	Internal consistency reliability coefficient
DEV	Academic skill development
DEV 1	First indicator measuring academic skill development
DEV 2	Second indicator measuring academic skill development
DEV 3	Third indicator measuring academic skill development
DEV 4	Fourth indicator measuring academic skill development
DEV 5	Fifth indicator measuring academic skill development
DEV 6	Sixth indicator measuring academic skill development
DEV 7	Seventh indicator measuring academic skill development
DEV 8	Eighth indicator measuring academic skill development
Doctoral	Educational background category
DV	Dependent variable
Educational Background	Demographic variable indicating undergraduate, postgraduate, or doctoral study level
Expert level	AI familiarity category indicating advanced self-reported knowledge of artificial intelligence
Female	Gender category
Field of Study	Demographic variable indicating respondents’ disciplinary area
Fornell-Larcker Criterion	Discriminant validity assessment comparing the square root of AVE with inter-construct correlations
F Change	Change in F statistic in the regression model
GFI	Goodness-of-fit index
GN	High-income country group
GS	Lower-to-middle-income country group
H1	Hypothesis that positive affect positively influences academic skill development
H2	Hypothesis that positive affect positively influences attitude toward ChatGPT
H3	Hypothesis that attitude toward chatgpt positively influences academic skill development
H4	Hypothesis that attitude toward chatgpt mediates the relationship between positive affect and academic skill development
H5	Hypothesis that learning orientation moderates the relationship between attitude toward chatgpt and academic skill development
H6	Hypothesis that learning orientation moderates the relationship between positive affect and academic skill development
High-income countries	Country income category representing respondents from high-income contexts
HTMT	Heterotrait–monotrait ratio
Independent Variable	Predictor variable in the model
IV	Independent variable
Learning Orientation	Moderating construct capturing mastery motivation, deep learning orientation, and orientation toward academic skill development
Lower-to-middle-income countries	Country income category representing respondents from lower-to-middle-income contexts
Male	Gender category
Max	Maximum observed value
Mean	Arithmetic average
Min	Minimum observed value
Moderately familiar	AI familiarity category indicating moderate knowledge of artificial intelligence
N	Number of observations
Natural Sciences	Field of study category in the demographic profile
NFI	Normed fit index
Not familiar at all	AI familiarity category indicating no self-reported familiarity with artificial intelligence
Original sample	Path coefficient estimate obtained from the original sample
*p*-value	Probability value used to assess statistical significance
PLEARN	Learning orientation
PLEARN 1	First indicator measuring learning orientation
PLEARN 2	Second indicator measuring learning orientation
PLEARN 3	Third indicator measuring learning orientation
PLS-SEM	Partial least squares structural equation modelling
POSEMO	Positive affect
POSEMO 1	First indicator measuring positive affect
POSEMO 2	Second indicator measuring positive affect
POSEMO 3	Third indicator measuring positive affect
POSEMO 4	Fourth indicator measuring positive affect
POSEMO 5	Fifth indicator measuring positive affect
Positive Affect	Independent variable capturing favourable emotional experiences during ChatGPT use, including enthusiasm, curiosity, excitement, engagement, and positive valence
Postgraduate	Educational background category
R	Correlation coefficient in the regression model
R^2^	Coefficient of determination
R^2^ change	Change in explained variance in the regression model
RMSEA	Root mean square error of approximation
RQ1	Research question examining how positive affect during ChatGPT use influences academic skill development
RQ2	Research question examining whether attitude toward ChatGPT mediates the relationship between positive affect and academic skill development
RQ3	Research question examining whether learning orientation moderates the affective and evaluative pathways to academic skill development
Sample Mean	Mean estimate obtained through bootstrapping
SAT	Satisfaction
SAT 1	First indicator measuring satisfaction
SAT 2	Second indicator measuring satisfaction
SAT 3	Third indicator measuring satisfaction
Satisfaction	Construct measuring students’ satisfaction with ChatGPT and its outputs
SD	Standard deviation
SE	Standard error
SEM	Structural equation modelling
Sig.	Statistical significance value
Social Sciences	Field of study category in the demographic profile
SRMR	Standardised root mean square residual
STDEV	Bootstrapped standard error
t-statistic	Test statistic used to assess path significance
Technology Acceptance Model	Theoretical model explaining technology acceptance through perceived usefulness, ease of use, attitude, and related outcomes
TLI	Tucker–Lewis index
Undergraduate	Educational background category
USE	ChatGPT use intensity
USE 1	First indicator measuring ChatGPT use intensity
USE 2	Second indicator measuring ChatGPT use intensity
USE 3	Third indicator measuring ChatGPT use intensity
USE 4	Fourth indicator measuring ChatGPT use intensity
USE 5	Fifth indicator measuring ChatGPT use intensity
Very familiar	AI familiarity category indicating high self-reported familiarity with artificial intelligence
